# Biogeochemical prospecting of metallic critical raw materials: soil to plant transfer in SW Ciudad Real Province, Spain

**DOI:** 10.1007/s11356-024-33097-0

**Published:** 2024-04-06

**Authors:** José Ignacio Barquero, Saturnino Lorenzo, Sofía Rivera, Ana C. González-Valoys, Efrén García-Ordiales, José María Esbrí, Pablo Higueras

**Affiliations:** 1 Instituto de Geología Aplicada, Universidad de Castilla-La Mancha, Pl. Manuel Meca 1, 13400 Almadén, Ciudad Real, Spain; 2 Escuela de Ingeniería Minera E Industrial de Almadén, 13400 Almadén, Ciudad Real, Spain; 3 IES Maestro Juan de Ávila, Ronda de Calatrava 1, Ciudad Real, Spain; 4 Facultad de Ingeniería Civil, Universidad Tecnológica de Panamá, Ricardo J. Alfaro Avenue, Dr. Víctor Levi Sasso University Campus, 0819-07289 Panamá City, Panamá; 5 ISYMAResearch Group, Mining, Energy and Materials Engineering School, University of Oviedo, C/ Independencia 13, 33011 Oviedo, Spain; 6 Departamento de Mineralogía y Petrología, Universidad Complutense de Madrid, Antonio Novais 12, 28040 Madrid, Spain

**Keywords:** Potentially toxic elements (PTEs), Bioavailability, Biogeochemistry, Holm oak (*Quercus ilex*), Bioaccumulation index, Plant-soil transfer, Sb

## Abstract

The soil–plant transfer of trace elements is a complex system in which many factors are involved such as the availability and bioavailability of elements in the soil, climate, pedological parameters, and the essential or toxic character of the elements. The present study proposes the evaluation of the use of multielement contents in vascular plants for prospecting ore deposits of trace elements of strategic interest for Europe. To accomplish this general goal, a study of the soil–plant transfer of major and trace elements using *Quercus ilex* as a study plant has been developed in the context of two geological domains with very different characteristics in geological terms and in the presence of ore deposits: the Almadén syncline for Hg and the Guadalmez syncline for Sb. The results have made it possible to differentiate geological domains not only in terms of individual elements, but also as a combination of major and trace elements using Factor Analysis. The bioconcentration factors have demonstrated the uptake of macronutrients and micronutrients in very high concentrations but these were barely dependent, or even independent of the concentrations in the soil, in addition to high values of this factor for Sb. The Factor Analysis allowed for the differentiation of geogenic elements from other linked to stibnite ore deposits (Sb, S, and Cu). This element (Sb) can be uptake by *Quercus ilex* via the root and from there translocating it to the leaves, showing a direct relation between concentrations in soil and plants. This finding opens the possibility of using *Quercus ilex* leaves for biogeochemical prospecting of geological domains or lithological types of interest to prospect for Sb deposits.

## Introduction

At present, society still depends on mineral resources coming from mining, influencing soil quality. In particular, the exploitation, processing, and transport of these metallic resources cause different alterations to the environment (Villadóniga et al. 2009). Those elements are generally referred to as “heavy metals” (HMs) or “potentially toxic elements” (PTEs) and produce highly adverse effects on the environment due to their persistence in soil, and their possible effects on its quality and on the health of living organisms, particularly plants (Macnicol and Beckett 1985) thus adding a layer of risk to the human food chain.

In recent years, the use of vegetal species as bioaccumulators or bioindicators has increased, as they allow the monitoring of PTE pollution, in particular in derelict mining areas, where these elements are an active source of contamination for ecosystems (Ugolini et al. 2013; Wang et al. 2019). Each different plant species possesses different bioaccumulation capacities for the various elements present in the soil (Liu et al. 2023). Suman et al. (2018) and Baker (1981) defined two main strategies adopted by plants which grow naturally on metalliferous sites based on the strategy of survival: “excluders,” representing the majority of plant species capable of surviving in soils containing elevated levels of PTEs, which have adopted the survival strategy of maximal exclusion of metal ions from the plant, and “hyperaccumulators,” which are plants that when exposed to elevated concentrations of PTEs are able to accumulate them in their above-ground parts without symptoms of phytotoxicity (Baker 1981; Rascio and Navari-Izzo 2011; Van der Ent et al. 2013).

Note that not all the higher-than-normal concentrations of elements in soils are related to mining or other anthropogenic activities; in some cases, they can be related to natural or geogenic factors (Rodrigues et al. 2009; Reimann and Garrett 2005).

The bioaccumulation capacity in various plant species depends not only on the physiology of the plant, but also on the speciation of the element in the soil, controlling its bioavailability (Maisto et al. 2004; Rossini Oliva and Mingorance 2004; Nagajyoti et al. 2010; Guzmán-Morales et al. 2011). Bioavailability of elements in the soil is a result of its physicochemical characteristics, such as reactivity (Bravo et al. 2017), clay and organic matter content, cationic exchange capacity, and the biochemical properties of litterfall (Alloway 2012; Birani et al. 2015; Teixeira et al. 2010).

On these bases, soil can be regarded as a biochemical reactor in which the concentrations of chemical elements depend on both natural and anthropogenic factors. Furthermore, natural factors can be classified as those related to the nature of the lithological substrate which condition the presence and abundance of different minerals providing different chemical elements, and factors related to the climatology and weathering of the lithological substrate, producing different processes, including hydrolysis and dissolutions, and causing the lixiviation and/or differential concentration of certain elements. These processes produce soils with different elemental composition (Adriano 2003). The anthropogenic activities, including spills, transferences, and the accumulation of residua, produce elemental changes considered to be contamination. Among them, mining and related activities constitute the most polluting processes, without being the only ones (Higueras et al. 2016).

Another concern to be taken into consideration in the elemental composition of soil is the chemical speciation of elements, which is directly related to their mobility and bioavailability (Shtangeeva et al. 2020; Nakazato et al. 2021). A significant proportion of the elements present in the soil are part of newly formed minerals, mostly in the form of hydroxides, but others transform into ionic forms, soluble in water, and are thus highly mobile and bioavailable (Augusto et al. 2017; Stein et al. 2017; Gerdol et al. 2018). Furthermore, these ionic forms can join other free charges of minerals such as clay, or else form macromolecular complexes with humus present in the soil; these correspond to “labile” elemental forms, available to plant uptake only under certain environmental conditions. Thus, the capacity of elemental plant uptake from the soil does not directly depend on their abundance, but on their “bioavailability,” produced by their presence in the soil in ionic or bioavailable forms.

The plant uptake of elements present in the soil occurs through the roots, being driven through the vascular system into the leaves where they accumulate (Augusto et al. 2017). The leaves also uptake and accumulate elements directly from the atmosphere, including elements contained in atmospheric particles (Monaci et al. 2000; Nagajyoti et al. 2010) and direct uptake of elements present in the atmosphere in vapor form, such as Hg (Barquero et al. 2019; Naharro et al. 2020).

Several studies (Rossini Oliva and Mingorance 2004; Guzmán-Morales et al. 2011; Hu et al. 2011; Zampieri et al. 2013; Song et al. 2015; Dafre-Martineli et al. 2020) have demonstrated that the elemental contents in vascular plants from diverse forestall ecosystems show an accumulation of metals on their leaves from mining and metallurgical activities. On this basis, biogeochemical characterization of an area should include the study of the distribution of chemical elements on their leaves and their variations as a reflection of the abundance of such elements in the soils, as well as, and to a higher degree, of their local bioavailability (Maisto et al. 2004). This data can be considered of high relevance as it can establish a relationship between the presence of PTEs in the plant and polluted soils (Monaci et al. 2022). To render this information more meaningful, the elemental analysis of soil allows the estimation of the “biological transference factor” or bioaccumulation factor (*BAF*), the ratio between the concentrations of the element in the soil and in the plant (when *BAF* > 1, bioconcentration occurs in the plant). Different plant species show various *BAFs*, and the same plant usually shows different *BAFs* for disparate chemical elements; therefore, if a species growing in a similar substrate presents varied *BAFs* in different areas, there is an indication of varied bioavailability for the element in those individual areas.

Toxicological problems are produced in mining areas related to the presence of PTEs that usually infiltrate the soil, transform into bioavailable forms, and undergo uptake by the vegetal cover (Alloway 2013). Most plant species uptake the PTEs present in mining-contaminated soils, and these are transferred to their aerial parts (Kumar et al. 1995).

**Fig. 1:**
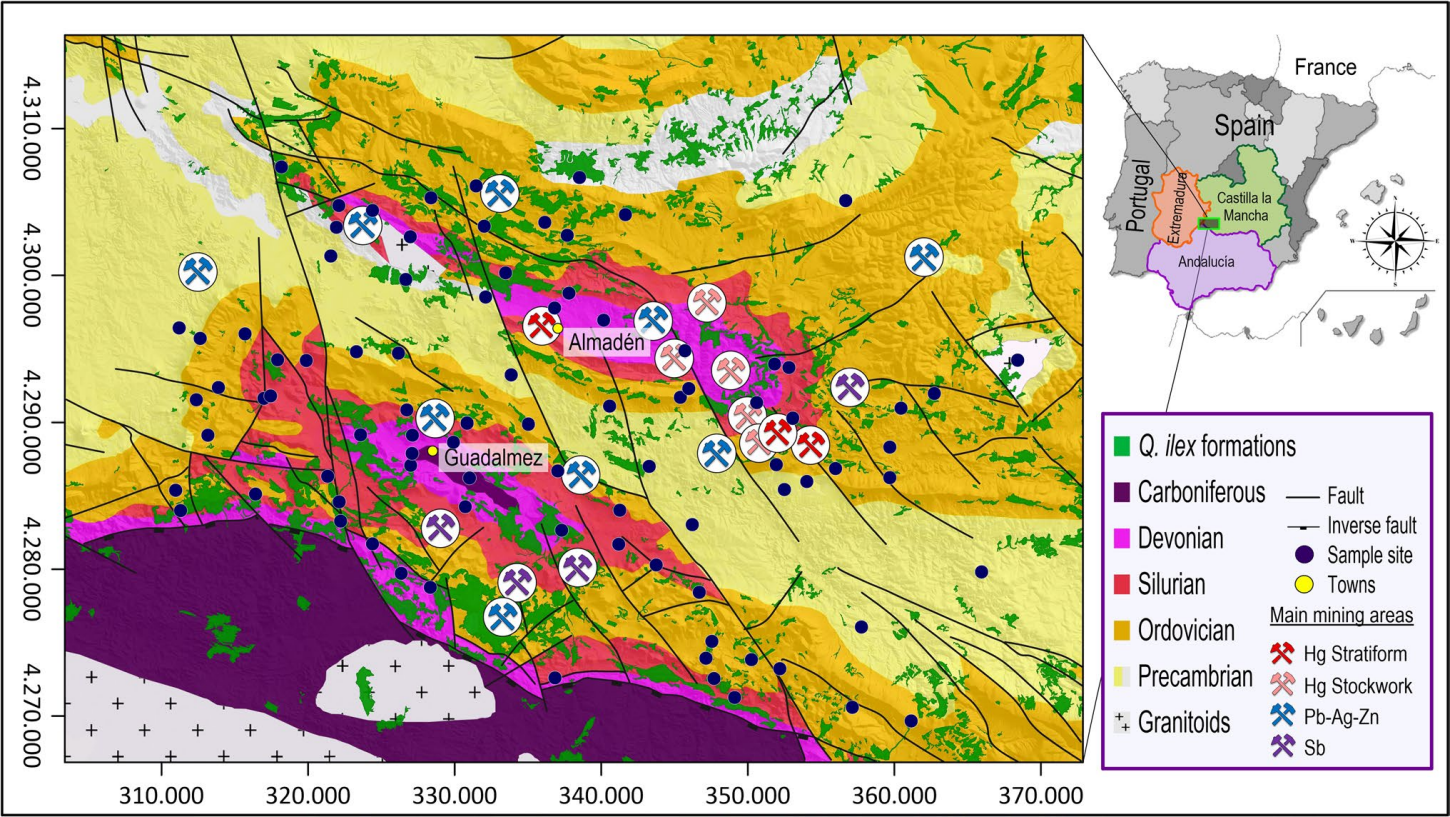
Sampling network in the syncline areas with a geological scheme and holm oak vegetation cover (*Q. Ilex*) formations. Geology modified from Clariana-García et al. (2022); mineral deposits locations taken from Gumiel and Arribas (1987), Hernández et al. (1999), and Palero et al. (2003); and distribution of *Q. ilex* formations taken from Villadóniga et al. (2009)

Although plants have been used in the prospection of mineral deposits, the usage was based more on the presence or absence of certain species, or effects of certain elements on their physiology, rather than on the bioaccumulated multielement contents, a more important aspect of environmental biogeochemistry than of biogeochemical prospecting. The present manuscript attempts to provide new data in this line of research that has been little studied in the literature. Therefore, the main objective of this study was to assess the influence of different types of lithological substrates, as well as the proximity of metallic mineralizations on the elemental composition of the leaves of *Quercus ilex* growing in an area intensely populated by this species. Moreover, an examination of the distribution of *BAF* was carried out in order to differentiate areas characterized by the different mobility of selected elements. To target these objectives, an extensive area was selected in the southern Central Iberian Zone from the Iberian Massif (San José et al. 1990), and in particular, three major geological structures: the Almadén and Guadalmez synclines and the Alcudia anticline (Fig. 1). Selection criteria were based on their quite heterogeneous geochemical characteristics between geological domains, and a notable number of decommissioned metal mines (Hg, Sb, Pb–Zn-Ag) disseminated in the study area (Gumiel and Arribas 1987; Saupé 1990; Hernández et al. 1999; Palero et al. 2003).

### Description of the study area

The studied area is located between the Ciudad Real (Castilla-La Mancha region) and Córdoba (Andalucía region) provinces, in south-central Spain. From the geological point of view, the area corresponds to the southern Central Iberian Zone of the Iberian Massif (Julivert et al. 1980; Díez Balda et al. 1990), and in particular, it comprises the Almadén and Guadalmez synclines and the Alcudia anticline. Both synclines are constituted by Palaeozoic (meta)sedimentary sequences starting with the well-known Armorican quartzite, of Lower Ordovician age, and including a very complete sequence of quartzites, quartzitic sandstones, shales, and very scarce carbonate-rock interbeddings, ending with Carboniferous-aged materials. It is also important to note the presence of igneous rocks, mostly mafic, in these sequences; Higueras et al. (2013) describe those present in the Almadén syncline as corresponding to two magmatic events, one constituted mostly by alkaline basalts (with minor differentiated varieties) and the other including subvolcanic diabases of tholeiitic affinity. In the Guadalmez syncline Lorenzo et al. (2005) mention the presence of diabases, most likely similar to the tholeiitic diabases from Almadén, as synthesized by Villaseca et al. (2022). These Palaeozoic (meta)sedimentary materials rest on angular discordancy on the so-called “*Complejo esquisto-grauváquico*,” a thick alternation of shales and greywackes of Preordovician age, including two different successions also separated by an angular discordancy. More details can be found in García Sansegundo et al. (1987) for the Almadén syncline and in Lorenzo et al. (2005) for the Guadalmez syncline.

The area is also characterized by the notable presence of metallic ore deposits, none of which are currently active: the Almadén syncline hosts the Almadén Mercury Mining District (AMMD), which has been the most important producer of this element worldwide (Saupé et al. 1990; Hernández et al. 1999); the Guadalmez syncline hosts some minor Pb–Zn and Sb vein-type deposits (Gumiel & Arribas 1987); and the Valle de Alcudia anticline hosts a large number of medium-importance Pb–Zn-Ag vein deposits, described by Palero et al. (2003). The Sb deposits in the Guadalmez syncline are presently the target of detailed studies due to a renewed interest in this element by the European Union, who consider it to be a “critical raw material” (UE 2020), and has promoted a number of studies concerning this element in the southern Central Iberian area (Álvarez-Ayuso et al. 2022; Barquero et al. 2022; Esbrí et al. 2023) in the context of the AUREOLE (tArgeting eU cRitical mEtals (Sb, W) and predictibility of Sb-As-Hg envirOnmentaL issuEs) project, funded by the European ERA-MIN3 program.

The area is characterized by a semi-mountainous relief and sits 500–650 m.a.s.l., with an 850-m.a.s.l. peak, corresponding to the Csa Köppen-Geiger climatic area (hot-summer Mediterranean climate); the regional hydric balance corresponds to rainy winters and springs, and dry summers, with an annual precipitation average of 518 mm, and with 963 mm of potential evapotranspiration. Average yearly temperature is 16.2 °C (Climate-Data.ORG 2022).

The forestry of the area is characterized by the conspicuous presence of holm oak (*Quercus ilex*), constituting dense Mediterranean forest formations with the presence of bush species including rosemary (*Rosmarinus officinalis*), rockrose (*Cistus* sp.), thyme (*Thymus* sp.), and lavender (*Lavandula stoechas*) (Villadóniga et al. 2009). Some areas correspond to the characteristic “*Dehesa*” landscape, characterized by a lower density of *Q. ilex*, the absence of bushes, and land usage for cattle breeding. *Q. ilex* corresponds to the most frequent vegetal species in the area, as well as in most of the Southwest of the Iberian Peninsula (Rafii et al. 1991; Silva 2007); this species was chosen as a model to accomplish the purpose of the present study. *Q. ilex* is a perennial tree belonging to the Fagaceae family. Furthermore, *Q. ilex* has frequently been used as a biomonitor of organic and inorganic pollutants present in the soil (Alfani et al. 2000; Orecchio 2007; Higueras et al. 2017).

De Nicola et al. (2013) used this species and epiphyte lichens to biomonitor air contaminants in Campania and Tuscany (Italy); the *Q. ilex* leaves showed a higher bioconcentration capacity, in particular for low molecular weight PTEs. Esposito et al. (2019) and Maisto et al. (2013) studied the elemental composition of the leaves of this species and soils, revealing high concentrations of Pb and Cu in areas affected by human activities with respect to background areas. Higueras et al. (2017) performed a similar study in the Pb–Zn-Ag mining district of Valle de Alcudia, analyzing Pb, Zn, Cu, As, Sb, Cd, and Hg, finding levels higher than the allowable toxicity threshold for Pb and Zn in *Q. ilex* leaves. As a general conclusion, *Q. ilex* has shown good possibilities to monitor contamination related to PTEs, but at issue here is if it can also be used to differentiate different substrates on which this species grows.

## Material and methods

### Sampling procedures

The sampling design was based on a soil grid with 150 sampling sites, including the collection of soil samples and of *Q. ilex* leaves, if a tree was present on the site; there were 88 leaf samples corresponding to the Almadén syncline (*N* = 29), Guadalmez syncline (*N* = 44), and Valle de Alcudia anticline (*N* = 15). Figure 1 shows the distribution of the samples together with the regional geology (adapted from Clariana-García et al. 2022), the location of the largest derelict mines (from several sources), and the location of the most important *Q. ilex* forest formations (taken from Villadóniga et al. 2009).

Leaf samples were taken all along years 2020 and 2021. Between one and four trees were sampled at each site, always corresponding to mature specimens (trunk diameter > 15–20 cm). Samples taking included some 60 leaves from each location, which were taken from all around the trees to obtain homogeneous and representative samples following the methodology suggested by Ernst (1995) for this type of survey. The leaves collected were also mature, older than 1 year, healthy, and not collected near the outermost branches. The leaves were collected using pruning shears, at > 2 m high above ground, and were stored in paper envelopes and transported to the laboratory. At the same site, a composite sample of 2 kg of soil was collected from the A horizon (topsoil, 0–15 cm) using an Ejkelkamp soil sampler (see Barquero et al. 2022 for more soil sampling details).

### Sample processing and chemical assay

Once in the laboratory, the leaves were washed with deionized water to eliminate surficial contamination by dust, in order to properly analyze the contaminants and chemical components taken from the soil (McCrimmon 1994; Alfani et al. 2000). Afterward, the leaves were dried in a laboratory oven prior to a trituration using a KINEMATICA mixer (MB800 B). A 5-g aliquot was then blended with 0.15 g of agglutinant (dissolution of Elvacite 2046 PANalytical and ACETONA PURISS (CH_3_(CO)CH_3_), UN 1090), inserted in aluminum vessels (RETSCH PP25) and compressed using a hydraulic hand press (SPECAC 250 kN), to obtain a pressed pill for use in the analytical procedure. Soil samples were air-dried at room temperature for 15 days and were then disaggregated, homogenized, and sieved to discharge the > 2-mm fraction; two aliquots were then obtained, one to determine the physicochemical parameters of the soil and the second was milled to < 100 µm in an automatic agate mortar (Barquero et al. 2022).

Analytical determinations of both types of samples (pressed pills of leaves and milled soil) were carried out by means of energy-dispersive X-ray fluorescence (EDXRF) using a Malvern-Panalytical Epsilon One device. Total Hg was analyzed on the milled samples using atomic absorption spectrometry with Zeeman effect and a high-frequency modulation of light (ZAAS-HFM) using a Lumex RA-915 M device with a pyrolytic unit (PYRO-915 +); this procedure involves the pyrolysis of the samples (both soil and vegetal) at 900 °C and their transport by filtered air to the analytical cell (Esbrí et al. 2021).

Several CRMs of soils (NIST 2710A) and plants (GC7162) were analyzed to check precision and accuracy. PTE recovery rates were in the range of 92–115% (EDXRF) for trace elements considered in this study and 95–102% for Hg (ZAAS-HFM).

### Soil to plant transfer indices

The bioaccumulation factor (*BAF*) was formulated (Eq. (1) as a simple parameter to assess the bioavailability of elements in the soil, and the capacity of the plant to uptake them (Inacio et al. 2014; Gruszecka-Kosowska 2019).

1 $$\left(BAF\right)= \frac{[{C}_{{\text{plant}}}]}{[{C}_{{\text{soil}}}]}$$

where [*C*_plant_] and [*C*_soil_] represent the concentration (in mg kg^−1^) of a given element in the leaves and soil, respectively, corresponding to the same sampling site. Values of *BAF* > 1 indicate a high bioaccumulation capacity, especially when *BAFs* are calculated with total concentration in soil. The *BAFs* calculated with the soluble fraction in soil are higher, although they more precisely express the hyperaccumulator condition of the plant in a certain polluted substrate.

### Statistical analysis and mapping

The analytical data was statistically treated using Minitab 19.1 software, aimed to determine both individual parameters (range, average, standard deviation, variation coefficient) and multielemental parameters (correlation coefficient, clustering, factor analysis). Moreover, and to unveil the geographic distribution of the analytical results, Surfer 21.1.158 (Golden Software) was employed to obtain distribution maps using Inverse Distance to a power as an interpolation method. Categorized maps used the 0–20%, 20–40%, 40–60%, 60–80%, and 80–100% percentiles to distinguish data populations.

With the objective of understanding the elemental distribution in the different geological domains and the different lithological substates, clustering and Factor Analysis were applied, obtaining the dendrograms, corresponding to Euclidean distance for the proximity type, and Ward’s for the agglomeration method, as well as the plots of factors 1 and 2.

## Results and discussion

### Multielemental concentrations of Quercus ilex leaves

The multielemental concentration data of *Quercus ilex* leaves obtained by EDXRF has a limitation in that a large part of the major elements (C, H, N, O) are not detectable by the available equipment (EDXRF) due to being too light to be properly quantified using this technique. Despite this, the major elements quantified included Ca, K, and Si, with 0.7, 0.4, and 0.2% on average, respectively (Table 1). A high content of Si can be explained by absorption from the siliceous soil as monosilicate acid or amorphous silica (Richmond and Sussman 2003) and are in the range of grasses and leguminous plants (< 1.2%) (Kabata-Pendias & Mukherjee 2007). Trace element concentrations demonstrated high contents of S, Mn, P, Fe, and Cl, with notable contents of some PTEs such as Cu, As, Sb, Pb, Hg, and Co. A noteworthy fact is that among these trace elements there is a group showing high variation coefficients (Zn, Pb, Hg) that coincide with the elements present in the ore deposits from the study area (Pb–Zn-Ag and Hg-Sb sulfides). In geochemical prospecting, high coefficients of variation suggest the existence of values outside the range of average + 2 standard deviation and are therefore interesting for the location of prospective areas. Surely the anomalous contents of these elements in the soils explains this transfer of non-essential elements to the *Q. ilex* specimens. However, Sb does not show high variation coefficients, probably due to the low mobility of this element in a semi-arid climate, as shown by Esbrí et al. (2023). Multielemental content data for holm oaks are not abundant in the scientific literature; thus, the works of Higueras et al. (2016) and Monaci et al (2022) were chosen as a comparison due to their similarities in the study area in physiographic and mineralogical terms. After a comparison of the dataset, in this study only Sb and Hg exceeded the contents published by Monaci et al. (2022), demonstrating the ability of the plant-species to capture Sb through the roots, bioaccumulate, and translocate to the above-ground parts of the plant, in addition to the Hg uptake directly from the atmosphere in gaseous form in an area deeply enriched for these elements, especially for Hg, as it has been commented in the “Introduction” section. Data from Higueras et al. (2016) belong to a small derelict Pb–Zn mine into the study area of this work. It can be observed that only Hg contents are significantly higher on average than the reference values (Higueras et al. 2016; Monaci et al. 2022), due to the absence of cinnabar mineralizations in the reference areas.

**Table 1:** Statistical summary of elemental concentrations in *Q. ilex* leaves from the study area

	Unit	Average	SD	VC	Range	Higueras et al. (2016)	Monaci et al. (2022)	Kabata-Pendias and Pendias (2001)
Si	%	0.2	0.1	48.8	0.1–0.6			
K	%	0.4	0.1	26.0	0.3–0.8		0.6	
Ca	%	0.7	0.2	23.7	0.4–1.4			
P	mg kg^−1^	746.3	100.3	13.4	466.3–997.9		800.0	
S	mg kg^−1^	1032.3	100.7	9.8	811.5–1360.0		1300.0	
Cl	mg kg^−1^	198.4	167.0	84.2	28.9–921.2			
Ti	mg kg^−1^	45.1	29.4	65.2	5.8–192.8			
Mn	mg kg^−1^	833.6	464.6	55.7	180.4–2670.0			
Fe	mg kg^−1^	322.9	163.7	50.7	78.6–1020.0		240.0	
Ni	mg kg^−1^	3.6	1.5	40.5	1.0–7.1		3.6	10–30
Cu	mg kg^−1^	7.2	1.3	18.8	5.2–14.1	4.6	6.1	10–30
Zn	mg kg^−1^	26.4	27.5	104.3	11.7–204.2	20.6	34.6	100–500
As	mg kg^−1^	0.4	0.2	52.3	0.1–1.2		0.4	1–20
Br	mg kg^−1^	3.6	1.4	38.0	0.6–6.6			
Rb	mg kg^−1^	2.5	1.1	42.4	0.5–7.5			
Sr	mg kg^−1^	12.7	4.5	35.1	4.6–25.5			
Zr	mg kg^−1^	2.2	0.9	41.2	0.5–6.5			
Sn	mg kg^−1^	15.2	1.2	7.7	12.0–18.3			
Sb	mg kg^−1^	3.6	0.8	23.1	1.2–5.6		0.1	
Te	mg kg^−1^	11.4	1.1	9.2	9.5–15.0			
Ba	mg kg^−1^	16.0	7.0	43.7	3.9–48.7		14.9	
Pb	mg kg^−1^	0.8	2.0	246.1	0.3–13.5	1.5	3.1	
Hg	ng g^−1^	113.9	169.6	148.9	10.6–1000.0	30	60	0.5–8
Co	mg kg^−1^	10.9	4.1	37.8	0.5–17.7			10–40

Reference values taken from Higueras et al. (2016) and Monaci et al. (2022), while growth depression values were adapted from Kabata-Pendias and Pendias (2001)

*SD*: standard deviation; *VC*: variation coefficient

**Fig. 2:**
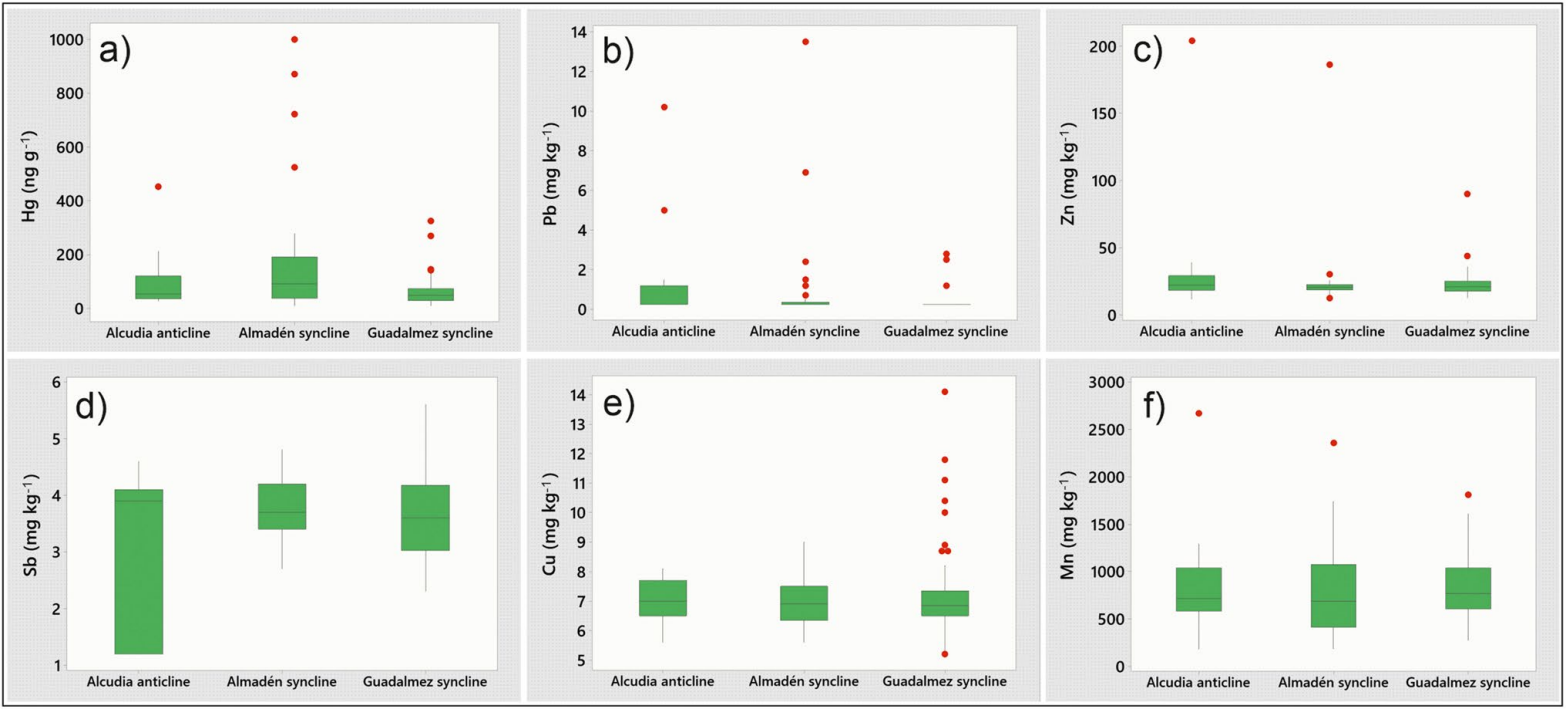
Boxplots of selected element concentrations in plants by geological domain. Outliers appear as red dots

Figure 2 shows the distribution of the concentrations of some elements of mining interest according to the geological domain to which they belong: the Alcudia anticline and the Almadén and Guadalmez synclines. As described by Barquero et al. (2022), there are clear differences in terms of the presence of metallic mineralizations between these domains, with Hg mineralizations predominating in the Almadén syncline, Sb in the Guadalmez syncline, and Pb–Zn in the three domains, although with a greater presence in the Alcudia anticline and with high concentrations of Cu in the Guadalmez syncline (Higueras et al. 2016). It can be observed that all the elements presented have similar averages in the three geological domains, although significant differences are evident in the Hg outliers in the Almadén syncline (Fig. 2a) and Cu in the Guadalmez syncline (Fig. 2e), corresponding to samples taken near possible small ore deposits not outcropping. It is necessary to highlight the absence of notable differences between domains for Sb (Fig. 2d), since it is an element which has been mined almost exclusively in the Guadalmez syncline, in addition to being an element with low mobility and very low bioavailability (Barquero et al. 2022). The most reasonable interpretation is that Sb levels in *Quercus* leaves correspond to local background levels for this element in these lithological units, slightly higher than the local background levels in the *Colline Metallifere* (Monaci et al. 2022). A similar behavior can be seen in Mn geological domain fractionation (Fig. 2f), with a similar average and distribution in the anticline and synclines. This is an element typically present in soils as oxides or oxydroxides, usually with a higher concentrations of soil trace elements but with expected differences in bioavailability depending on pH and redox potential (Kabata-Pendias & Mukherjee 2007).

**Fig. 3:**
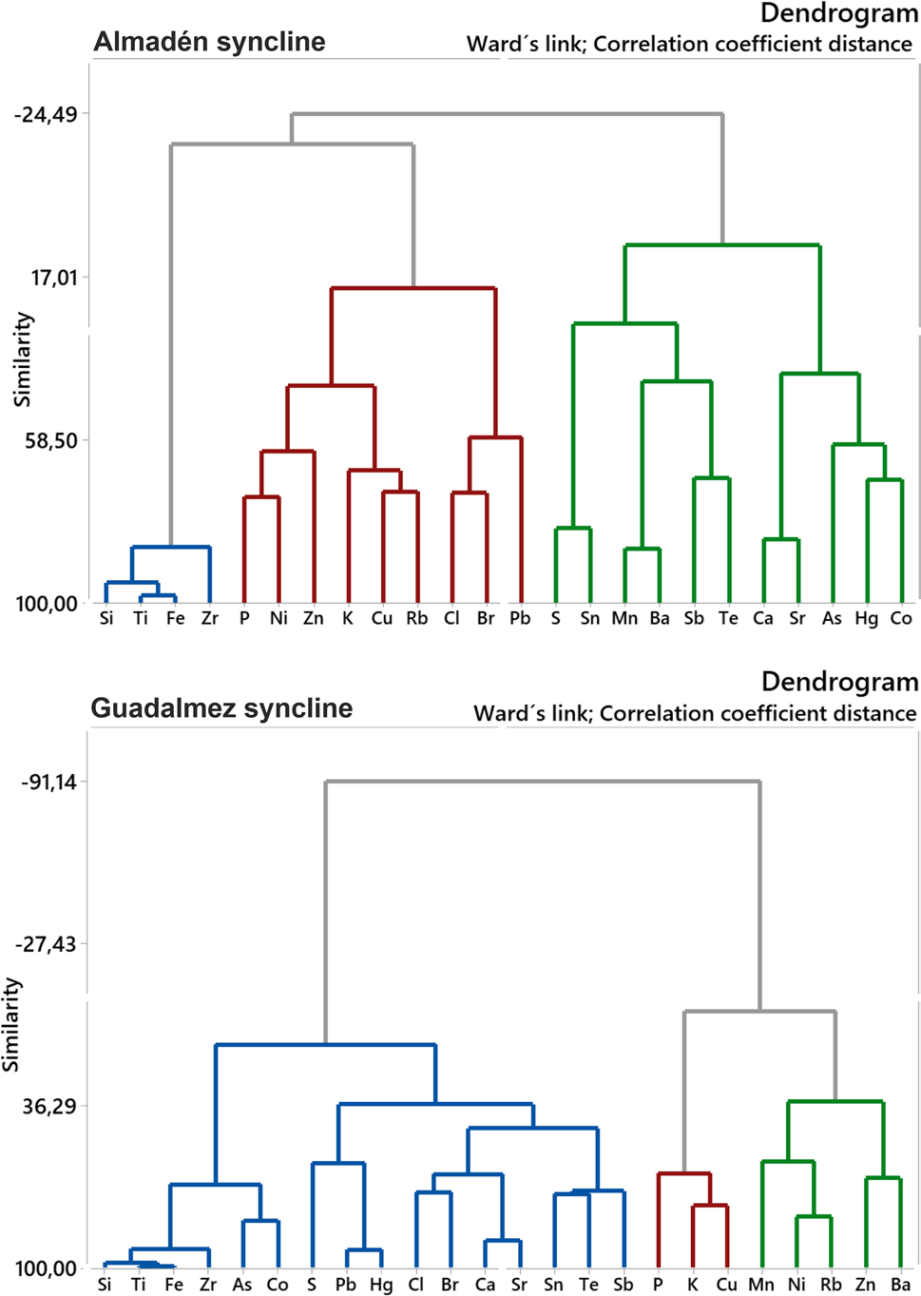
Dendrograms of elemental results by syncline

As displayed in Fig. 3, notable differences were found in the formation of clusters of elements between both synclines: Almadén (Hg) and Guadalmez (Sb). No clear differentiation has been found between major and trace elements in their transfer or incorporation from the substrate to the soil. In the Almadén syncline, the cluster related to Hg uptake also includes major elements such as Ca (related to volcanic activity), and other elements related to mineralized zones such as As, Co, or S. The lithological (or geogenic) cluster which includes Si, Ti, and Fe and this group appears to be a subgroup of some essential elements for plants (P, Zn, K, Rb, Zn, Cl, Br) but clearly differs from the group related to syncline mineralizations.

In the Guadalmez syncline, however, the geogenic cluster is included as a subgroup together with that related to Sb mineralizations, and it is very clearly differentiated from another cluster made up of essential elements for plants, such as P, K, Mn, Rb, Zn, and Ba.

This differentiation may be due to differences in the mineralizations of Hg and Sb, which appear exclusively related to quartzites only in the Guadalmez syncline for Sb; meanwhile, in the Almadén syncline the “Las Cuevas”-type Hg deposits may appear in a wider variety of lithologies.

### Soil to plant transfer indices

The usage of the *BAF* (Alloway et al. 2012) has demonstrated a generalized low bioconcentration capacity for *Q. ilex*, this parameter being lower than unity for most of the elements considered: only P, S, K, Ca, Mn, Sr, Sb, and Co show an average *BAF* > 1, followed by Ti, Ni, Cu, Zn, and Mo, with a maximum *BAF* > 1 (1.3–3.9).

Table 2 shows the *BAF* values classified according to the need/ability to uptake particular elements from the soil. The elements with the highest *BAF* values are, as expected, the macronutrients (primary and secondary), which show averages greater than 1 in all cases and with very high maximums, especially in elements such as S and Ca, present in the soils of both synclines and available to the plants. In the group of micronutrients, only Co and Mn exhibit *BAF* averages greater than 1, although there are discrete values above this threshold for some other elements (Cu, Mo, and Zn) that could reach toxic concentrations for the plant. Some of these elements (Cu and Zn) have a variety of possible sources such as the background content, depending on the lithology, sulfide ores, and phytosanitary products. Other elements are common but not essential for plants, like Sr, with average values of *BAF* > 1, and Ti, with some high *BAF* values reaching a maximum of 3.9. Finally, in the group of elements that can be toxic to the plant, it is necessary to mention the high *BAF* values (on average) for Sb, with maximums reaching 5.6 in some areas. The significance of this high *BAF* value may not be relevant, since it has a very low coefficient of variation and the Sb contents in soil and plant are close to the detection limit of the EDXRF equipment. The coefficient of variation can be indicative of differences between the sampling points in terms of the mobility of the element, depending on its lithological composition and/or presence of ore deposits. There are several elements with coefficients of variation greater than 1, a micronutrient (Co), some common elements (Mn, V), and others that can be toxic for plants (Pb, Hg, Cr, and Al).

**Table 2:** Statistical summary of *BAFs* in *Q. ilex* leaves from the study area

	Average	SD	VC	Range	Esposito et al. (2019)	Maisto et al. (2004)	Higueras et al. (2017)
*Primary macronutrients*							
P	**2.3**	0.9	0.4	(0.4–**4.6)**			
K	**4.3**	2.4	0.5	(1.1–**13.6)**			
*Secondary macronutrients*							
S	**12.3**	5.5	0.4	(3.4–**27.2)**			
Ca	**8.2**	6.0	0.7	(0.5–**39.8)**			
*Micronutrients*							
Co	**1.5**	1.9	1.3	(0.0–**12.2)**			
Cu	0.6	0.5	0.7	(0.1–**2.6)**	0.10	0.12–0.18	0.10 ± 0.12
Fe	0.0	0.0	0.7	(0.0–0.0)			
Mn	**2.4**	3.0	1.3	(0.1–**18.9)**			
Mo	1.0	0.6	0.7	(0.0–**2.6)**			
Zn	0.7	0.7	0.9	(0.1–**3.3)**			0.15 ± 0.13
*Common elements in plants*							
Si	0.0	0.0	0.5	(0.0–0.0)			
Ti	0.6	0.5	0.8	(0.0–**3.9)**			
Sr	**1.2**	0.6	0.5	(0.2–**2.9)**			
Ba	0.2	0.1	0.6	(0.0–0.5)			
V	0.0	0.0	2.2	(0.0–0.2)			
*Toxic elements for plants*							
Ni	0.3	0.3	0.9	(0.0–**1.3)**	0.12		
As	0.0	0.0	0.8	(0.0–0.2)			
Pb	0.0	0.1	3.1	(0.0–0.6)	0.04	0.01–0.02	0.02 ± 0.05
Hg	0.1	0.1	1.0	(0.0–0.9)			0.35 ± 0.90
Cr	0.1	0.1	1.4	(0.0–0.6)	0.05	0.01–0.05	
Sb	**2.2**	1.5	0.7	(0.0–**5.6)**			
Al	0.0	0.0	2.7	(0.0–0.1)			

Values of *BAF* > 1 appear in bold in average and maximum columns

*SD*: standard deviation; *VC*: variation coefficient

**Fig. 4:**
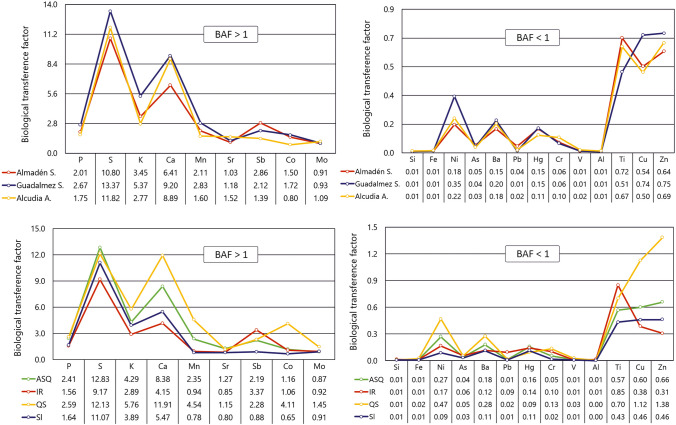
Plots of *BAFs* by geological domains (**A** and **B**) and by type of geological substrate (**C** and **D**). Abbreviations: ASQ: alternations of shales and quartzites, IR: igneous rocks, QS: quartzites and sandstones, Sl: slates

**Fig. 5:**
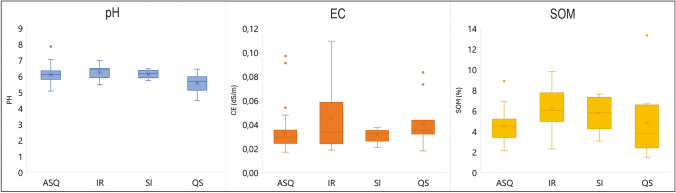
Distribution plots of pH, electrical conductivity (EC), and soil organic matter (SOM) by lithological substrates

On the other hand, the influence of the geological substrate must play a role in plant uptake. Figure 4 displays this relationship to geological domain (Fig. 4A, B) and lithological domain (Fig. 4C, D). Elements showing *BAF* > 1 seem to have a greater capacity to bioaccumulate the macronutrients in the Guadalmez syncline located further south in an area with typically Appalachian relief and forest-like vegetation. This trend can also be observed for other elements with *BAFs* < 1, including Ni, Ba, Hg, Cu, and Zn. All these higher *BAFs* in the Guadalmez syncline are probably related to the lower water stress of *Quercus* trees in this area, as according to the National Atlas of Spain this area is located in the southern limit of a climatic zone with lower maximum temperatures in summer, higher minimum temperatures in winter, and similar rainfall (IGN Clima 2022). However, the differences by lithological substrates are not particularly evident, although a certain predominance of the quartzite substrate (QS in Fig. 4 C and D) is observed for some elements (K, Ca, Mn, Co, Ni, Ba, Cu, and Zn), and notably high *BAFs* in the mafic igneous rock (IR) unit for the elements of greatest mining interest such as Sb, Pb, and Hg. Moreover, the acid reactivity in soils developed in the quartzite units is contrastingly higher, with pH significantly lower than in the rest of the lithologies (Fig. 5). Moreover, it is noteworthy that the generalized low *BAFs* were found on shaly substrates where the weathering should be more active, releasing the elemental composition much more easily than in quartzite-dominated substrates.

### Factor analysis of elemental composition

**Fig. 6:**
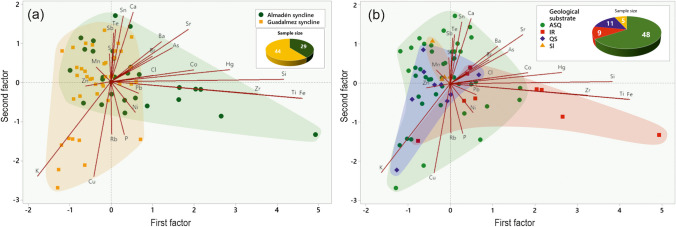
Factor analysis plots, separating samples by synclines (**a**) and lithological types (**b**)

The Factor Analysis shown in Fig. 6a clearly distinguishes groups of samples by synclines, with factor 1 (strongly influenced by the variations in Hg concentration) determining this distinction in the case of the Almadén syncline and factor 2 (dependent on Sb concentrations) determinant for the Guadalmez syncline. There are some more elements in both factors: factor 1 includes geogenic elements (Si, Ti, Fe, Zr, Mn), along with metals from the most ubiquitous mineralizations throughout the study area (Pb and Zn), and factor 2, however, includes Sb and elements linked to the stibnite mineralizations (S and Cu).

However, grouping by lithological types (Fig. 6b) does not offer a clear distinction with a totally undifferentiated group (ASQ), the group of igneous rocks clearly dependent on factor 1 (a lithological type widely represented in the Almadén syncline), and the other two lithological groups dependent on factor 2, QS being the preferential lithological type for Sb ore deposits, as expected, since the stibnite mineralization are preferently hosted in quartzitic units.

These results show that the multielemental composition of *Q. ilex* leaves cannot be differentiated in terms of the concentrations groups micronutrients/macronutrients, since the plant uptakes what it needs from these elements, regardless of their existing concentrations in the soil. On the other hand, the trace elements that may be toxic to plants make it possible to differentiate samples by geological domains (synclines) and even by lithological types, since the plant accumulates these elements depending on the amount present and availability in the soil. In this way, it might be possible to use the concentrations of trace elements in leaves to prospect over large areas, geological domains, or lithological types, in which *Quercus* trees have a sufficiently representative distribution. These starting conditions could allow of finding ore deposits, at least of Hg and Sb, the two elements studied in the present work. This application of vascular plants to the biogeochemical prospecting of ore deposits would be especially useful for elements with very low mobilities and little leachable capacity such as Sb, which makes it difficult to prospect in soils and sediments, but less useful for elements such as Hg, highly volatile and mobile, prospectable in soils, sediments, waters, and even in the atmosphere. The application of biogeochemical prospecting for Sb is of particular current interest as it is an element which has been declared a critical raw material by the European Union and the prospecting of its deposits is a priority for the European continent (UE 2020). Given the low mobility of this element in the typical semi-arid climates of the Iberian Peninsula (Esbrí et al. 2023), the use of *Quercus ilex* leaves could delimit geological or lithological domains in which to focus prospecting efforts in other matrices (soils or sediments) or techniques (geological, geophysical, machine learning, etc.).

## Conclusions

In the studied Almadén and Guadalmez synclines, soil–plant transfer has been described using the leaves of *Quercus ilex*, the most ubiquitous tree in the area. The contents of major elements linked to macronutrients and micronutrients have been described, with high concentrations of Si, P, Ca, S, Mn, and Fe. The contents of trace elements (Cu, As, Sb, Pb, Hg, and Co) have shown significant concentrations, especially high in Sb and Hg (3.6 and 113.9 mg kg^−1^ on average). It was possible to differentiate groups of *Quercus ilex* samples according to geological domains and even lithological types. This differentiation was possible for some major elements linked to lithological types, such as Ca in the igneous rocks of the Almadén syncline, as well as for trace elements linked to the main ore deposits in the area (Hg and Sb). The results have also shown very high *BAF* values for those elements that are macronutrients or micronutrients for the plant, also being significantly high for Sb (*BAF* = 2.2), a critical raw material of paramount importance for the European economy. After a factor analysis, it was possible to verify that the differentiation of lithological types and geological domains is highly dependent on the transfer of trace elements from the soil to the plant, being especially dependent on the Sb content in *Quercus ilex* leaves. This general conclusion opens the possibility to use of *Quercus ilex* in the biogeochemical prospecting of Sb applicable to large areas, extensive geological domains, or lithological types. The combination of these techniques as preliminary methods, in combination with other geochemical techniques in soil/sediments, geological or geophysical, may facilitate the location of new Sb deposits in the Iberian Peninsula. In the realm of ore prospecting, deriving meaningful insights from disjointed and contextually incomplete data poses a significant challenge, particularly when dealing with soil or sediment data lacking connection to the broader geological context. Furthermore, the utilization of evidence, albeit occasionally subtle, extracted from such datasets often fails to integrate with complementary geophysical information, hindering the delineation of prospective regions. Therefore, adopting a holistic approach that incorporates data on bioaccumulation in vascular plants could enhance the identification of mineral deposits containing critical raw materials previously undetectable via conventional methodologies. This approach should be tested in nearby areas with lithological differences but presence of similar plant species, such as in the Iberian pyritic belt.

## Data Availability

The data that support the findings of this study are available from the corresponding author, [jesbri@ucm.es], upon reasonable request.
